# Having an Old Friend for Dinner: The Interplay between Apoptotic Cells and Efferocytes

**DOI:** 10.3390/cells10051265

**Published:** 2021-05-20

**Authors:** Austin Le Lam, Bryan Heit

**Affiliations:** 1 Department of Microbiology and Immunology and the Center for Human Immunology, Schulich School of Medicine and Dentistry, Western University, London, ON N6A 5C1, Canada; alam337@uwo.ca; 2 Robarts Research Institute, London, ON N6A 3K7, Canada

**Keywords:** efferocytosis, cell death, apoptosis, intracellular trafficking, transcriptional regulation, cellular metabolism, inflammation, resolution

## Abstract

Apoptosis, the programmed and intentional death of senescent, damaged, or otherwise superfluous cells, is the natural end-point for most cells within multicellular organisms. Apoptotic cells are not inherently damaging, but if left unattended, they can lyse through secondary necrosis. The resulting release of intracellular contents drives inflammation in the surrounding tissue and can lead to autoimmunity. These negative consequences of secondary necrosis are avoided by efferocytosis—the phagocytic clearance of apoptotic cells. Efferocytosis is a product of both apoptotic cells and efferocyte mechanisms, which cooperate to ensure the rapid and complete removal of apoptotic cells. Herein, we review the processes used by apoptotic cells to ensure their timely removal, and the receptors, signaling, and cellular processes used by efferocytes for efferocytosis, with a focus on the receptors and signaling driving this process.

## 1. Find Me and Eat Me Signals: Apoptotic Cells Write Their Own Menu

**Figure 1:**
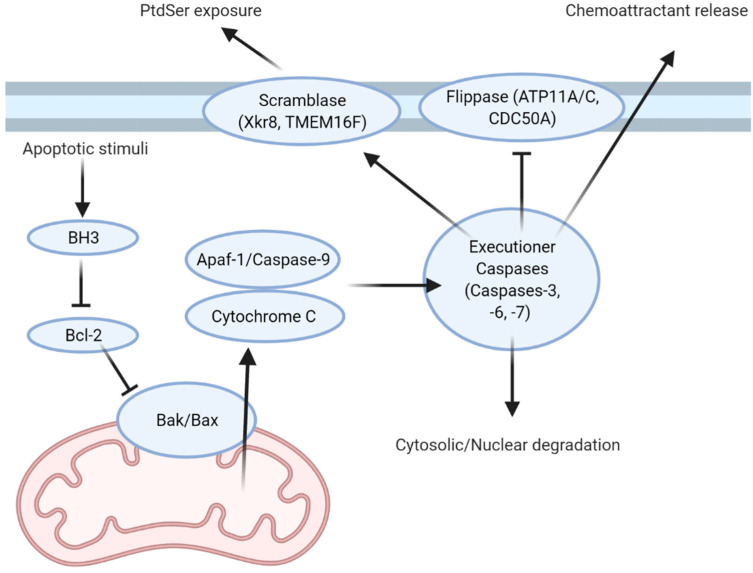
General scheme of apoptotic signaling in mammals. Apoptotic stimuli initiate signaling cascades that converge on the activation of BH3 domain-containing proteins. Activation of these proteins inhibits anti-apoptotic proteins such as Bcl-2, resulting in the oligomerization of the BAK/BAX complex in the outer mitochondrial membrane. BAK/BAX oligomerization forms a pore which allows for the release of cytochrome C into the cytosol, where it nucleates the formation of the Apaf1/caspase-9 apoptosome. The apoptosome catalyzes the activation of executioner caspases (caspase-3, -6, and -7), which are responsible for mediating the disassembly of the apoptosing cell. In addition to driving the degradation of the apoptotic cell, caspase-mediated cleavage also induces PtdSer exposure through the combined inactivation of flippases and the formation of constitutively active scramblases, and also induces the release of “find-me” signals via a variety of mechanisms.

Apoptosis has been extensively studied in the model organism *Caenorhabditis elegans*, where the cell number of the organism is stringently regulated [1,2,3,4,5,6]. *C. elegans* regulates cell death through programmed cell death proteins called CEDs, with orthologs of these proteins regulating apoptosis in other multicellular organisms. In mammals, apoptotic stimuli activate a number of apoptotic pathways, with these pathways converging on the inhibition of Bcl-2, a paralogue of CED-9 [2,7,8,9,10]. Bcl-2 and its homologs in the B cell lymphoma (Bcl) family of proteins play antagonistic roles in regulating apoptosis through inhibiting the apoptotic effectors BAX and BAK [2,9,11]. Loss of this inhibitory signal enables oligomerization of BAX and BAK within the outer mitochondrial membrane, forming a pore which allows for the efflux of cytochrome C into the cytoplasm. Here, cytochrome C associates with caspase-9 and Apaf-1 to form a heptameric apoptosome complex [2,9]. This point marks the first irreversible step in apoptosis, after which the cell is committed to its own death. The apoptosome cleaves and activates the pro-forms of executioner caspases (caspases-3, -6, and -7; Figure 1). Once activated, the executioner caspases degrade cytosolic and nuclear components, as well as cleaving and activating a range of enzymes which further drive disassembly of the cell. This brings about the hallmark characteristics of apoptosis: nuclear fragmentation and condensation, membrane blebbing, and cleavage of cytosolic proteins [3,12,13,14,15,16]. These cytosolic materials are pro-inflammatory and potentially immunogenic, and therefore are contained within the cytosol of the apoptosing cell. However, during apoptosis, cellular energetics cease, putting a finite limit on the length of time for which these materials can be contained.

During the executioner phase of apoptosis, changes occur to the cell’s physiology and plasma membrane that promote recognition and clearance by efferocytic cells such as macrophages. This process can be divided into three steps: (1) recruitment of efferocytes (efferocytic cells), (2) recognition of the apoptotic cell, and (3) the engulfment and degradation of the apoptotic cell by the efferocyte. To garner the attention of remote phagocytes, apoptosis induces the release of chemoattractants: “find-me” signals which diffuse into the tissue surrounding the apoptosing cell [5,8,13,17,18,19]. This forms a concentration gradient which efferocytes can use to direct their movement towards the apoptotic cell. Often, these chemoattractants also carry out a secondary role in immune regulation, in addition to acting as a directional migratory signal.

Fractalkine, otherwise known as CX3CL1, is released during apoptosis in a caspase-dependent mechanism from some immune cells, [2,19,20,21,22]. Free CX3CL1 then promotes macrophage chemotaxis through the chemokine receptor CX3CR1. Deletions in the chemokine receptor result in impaired macrophage trafficking to the site of fractalkine release [23,24]. In microglia, fractalkine plays an additional role in the upregulation of MFG-E8, a phosphatidylserine (PtdSer) opsonin for efferocytic integrins, which will be discussed in a later section [5,25,26,27]. In addition, fractalkine dampens the neurotoxic effects of microglia-mediated efferocytosis of damaged neuronal tissue by inducing the production of anti-inflammatory cytokines [25,26].

Another chemoattractant, lysophosphatidylcholine (LPC) is produced upon the caspase-3-mediated cleavage and activation of calcium-independent phospholipase A_2_ [28,29]. Activated phospholipase A_2_ catalyzes the production of lysophospholipids, including LPC. Work by Murakami *et al*. and Peter *et al*. revealed that the G-protein-coupled receptor G2A is responsible for LPC recognition and subsequent efferocyte chemotaxis [18,30]. G2A knockouts have an autoimmune phenotype similar to systemic lupus erythematosus (SLE)—an autoimmune disorder driven, in part, by uncleared apoptotic cells—highlighting the importance of the LPC–G2A interaction for apoptotic cell clearance [13,31]. Unlike CX3CL1, which is only produced by a limited subset of apoptosing immune cells, LPC is a universal “find-me” signal produced by all apoptotic cells [29].

Sphingosine-1-phosphate (S1P) is another important apoptosis-related chemoattractant. S1P is normally restricted to the cytosolic leaflet of the cell membrane due to its production by sphingosine kinase 1 (SphK1) [17]. However, the SphK1 paralog SphK2 becomes active during apoptosis and produces large quantities of S1P on the extracellular leaflet of the plasma membrane [32,33]. Inhibition of SphK2 results in a lack of S1P production during apoptosis and delayed apoptotic cell clearance. The production of S1P during apoptosis requires the caspase-1 mediated cleavage of SphK2′s *N*-terminus, resulting in a constitutively active form [33,34]. Through an unidentified mechanism, cleaved SphK2 is released into the extracellular milieu, where, via a PtdSer binding domain, Sphk2 localizes PtdSer to the outer leaflet of the apoptosing cell. SphK2 activity is impaired following either the reduction of extracellular PtdSer exposure or mutations in its PtdSer binding domain [33]. Chemotaxis to S1P is mediated by a family of five GPCRs, dubbed the S1P receptors [35]. Extracellular S1P receptor activation also serves an additional role in efferocytosis through inducing the upregulation of erythropoietin, which acts in an autocrine fashion to promote efferocytosis [36]. Obstructing this pathway leads to impaired apoptotic cell engulfment and the expression of inflammatory cytokines.

The final major class of “find-me” signals are nucleotides such as ATP and UTP. These nucleotides are released during apoptosis following caspase-3-mediated cleavage of the Pannexin-1 channel [37,38,39]. Cleavage of Pannexin-1′s *C*-terminus exposes the channel’s pore, allowing the unregulated release of metabolites, including nucleotides. Extracellular nucleotides are recognized by nearby efferocytes via the purinoreceptor P2X7R and the chemotactic GPCR P2Y2 [40,41]. Preventing the release of nucleotides does not impair apoptosis but does hinder the recruitment of efferocytes such as macrophages. Conversely, P2X7R knockout results in impaired macrophage recruitment and enhancement of diseases associated with defective efferocytosis such as atherosclerosis [42].

Aside from releasing “find-me” molecules to attract efferocytes, apoptotic cells must also present themselves in a fashion that promotes their recognition and clearance through the exposure of “eat-me” signals and the loss of antagonistic “don’t-eat-me” signals. PtdSer is the primary eat-me signal used for recognition and engulfment by efferocytes [16,43]. Normally, PtdSer is restricted to the inner leaflet of the plasma membrane and therefore is not exposed to the extracellular environment in non-apoptotic cells. This polarized distribution is maintained by flippases, floppases, and scramblases, which are transporters that shuttle phospholipids from one leaflet of the membrane to the other [11,43,44,45,46]. Flippases are involved in maintaining PtdSer in the inner leaflet through the inward shuttling of phospholipids. Floppases have an antagonistic role and shuttle phospholipids outward, while scramblases facilitate the bidirectional transport of phospholipids [43,45,47,48,49]. Normally, the flippases ATP11C and CDC50A maintain PtdSer in the inner leaflet [43,46]. However, ATP11C possesses a caspase recognition site, which, when cleaved by active caspases, inactivates the flippase [43]. Mutations in the caspase cleavage sites yield a caspase-resistant ATP11C and prevents PtdSer exposure during apoptosis. Conversely, caspase-mediated cleavage of the scramblase Xkr8 produces a constitutively active form [44,49]. Active Xkr8 forms a homodimer that mediates the energy- and Ca^2+^- independent scrambling of PtdSer and phosphatidylethanolamine—another lipid normally restricted to the inner leaflet of the plasma membrane—to both leaflets. Like ATP11C, a lack of Xkr8 function results in impaired PtdSer exposure and is linked with autoimmune diseases such as SLE [50]. Concurrent loss of flippase activity and activation of scramblase activity result in the rapid accumulation of PtdSer in the outer leaflet of apoptotic cells. While other PtdSer scramblases exist, they do not appear to be involved in apoptosis. For example, TMEM16F is expressed in platelets and is activated by calcium-mediated signaling, where PtdSer exposure is require for platelet adhesion within a thrombus [48,51,52]. Additional “eat-me” signals have been identified, but their contribution to efferocytosis appears to be small and their release pathways and receptors are not as well characterized as PtdSer. As one example, calreticulin, a chaperone normally restricted to the endoplasmic reticulum, is exocytosed during apoptosis, where it binds to the membrane of the apoptotic cell. Here, it is bound by the efferocyte receptor LRP-1, which then mediates its engulfment [53].

In vivo, the presence of apoptotic cells is transient, indicating that efferocytosis is both rapid and efficient. Indeed, there is no “off switch” to apoptosis once executioner caspases have been activated, meaning that the loss of both cellular energetics and plasma membrane integrity are inevitable if an apoptotic cell is left uncleared, leading to its lysis [1,14]. This lytic process is termed secondary necrosis, in which uncleared apoptotic cells undergo cytosolic swelling and the progressive loss of membrane integrity, to the point where the cell ultimately lyses and releases its intracellular contents into the surrounding extracellular milieu. Secondary necrosis has been shown to induce inflammation as a result of the inflammatory and immunogenic molecules, including autoantigens and danger-associated molecular patterns, which are released from apoptotic cells undergoing secondary necrosis [1,54]. This release of autoantigens and proinflammatory compounds can lead to inappropriate immune cell activation, thereby driving inflammatory diseases such as atherosclerosis, and autoimmune disorders such as SLE and multiple sclerosis, topics covered in more depth later in this review [7,55,56,57,58,59,60].

## 2. Tasting the Apoptotic Cell: Efferocytic Receptors

**Figure 2:**
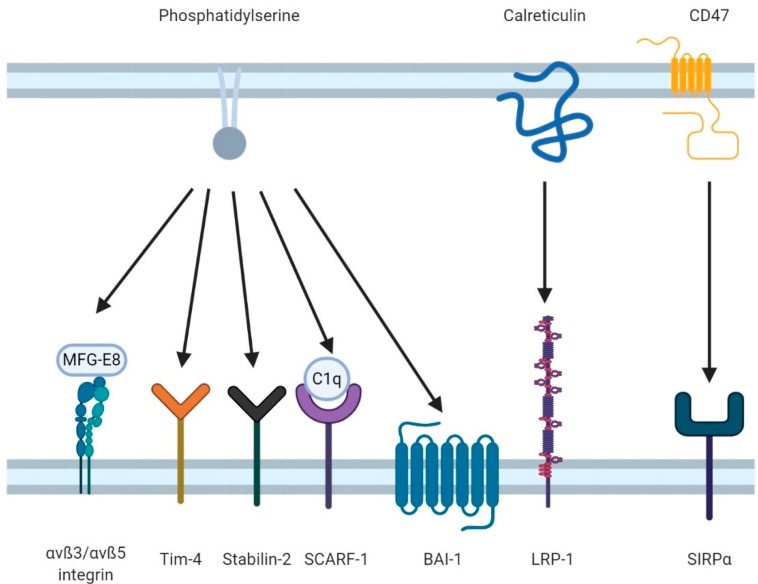
The induction of efferocytosis requires two signals: presentation of “eat-me” signals and loss of “don’t-eat-me” signals. Apoptotic cells are recognized by a variety of receptors through various ligands presented on their cell surface. PtdSer is the most common ligand and is bound both directly by receptors and indirectly via opsonins such as MFG-E8. Other apoptotic cell ligands include calreticulin, which normally resides within the endoplasmic reticulum. Opposing efferocytosis are the “don’t-eat-me” signals such as CD47, which, via SIRPα on the efferocyte, inhibit the signaling of efferocytic receptors.

Having migrated to the site of apoptosis through chemoattractant “find-me” signals, the efferocyte must next recognize and engulf the apoptotic cell. This engulfment occurs through a mechanism similar to the engulfment of pathogens during phagocytosis, but which relies on a different set of apoptotic cell-recognizing receptors. There are at least 10 known efferocytic receptors, some of which bind directly to apoptotic cells and some which rely on opsonins to act as “bridges” between the apoptotic cell and the efferocytes (Figure 2). Once bound, these receptors signal via a signaling pathway similar to the canonical phagocytic signaling pathway to mediate apoptotic cell engulfment [61,62,63]. As mentioned previously, the most common ligand for these receptors is PtdSer. It may appear redundant to have several receptors recognizing the same ligand, but extensive work has demonstrated that this heterogenous array of receptors serves important roles in efferocytosis, either through tissue-specific expression, or through induction of receptor-specific signaling and transcriptional programs (Table 1) [61,64]. Indeed, expression of these receptors varies greatly across tissues and across professional phagocytes—which, in most tissues, function as the predominant efferocyte—versus expression in tissues such as the kidneys, where non-professional phagocytes such as epithelial cells are the primary efferocytic cell types (reviewed recently by Boada-Romero *et al*. [65]). As the signaling of some of these receptors is poorly understood, this review will concentrate on the best understood efferocytic receptors: BAI-1, TIM-4, Stabilin-2, SCARF-1, integrins, and the TAM (Tyro3, Axl, MERTK) family of receptors.

The engulfment of apoptotic cells by efferocytes requires two independent signals, both which are generated as part of the apoptotic process. The first signal is the loss of inhibitory signaling (otherwise referred to as the “don’t eat me” signal), which acts as a tonic inhibitor of efferocytosis [98]. This activity is mediated by the recognition of CD47 on the plasma membranes of living cells. Normally, CD47 exists as microclusters within the cell membrane; these puncta enable CD47 to cross-link and activate their cognate receptor, SIRPα, on the efferocyte [98,99]. This engagement of SIRPα induces downstream inhibitory signaling through its ITIM motifs, thereby inhibiting efferocytic signaling in the efferocyte. These CD47 puncta are generated by anchoring of CD47 to the underlying actin cytoskeleton [70]. During apoptosis, caspase-mediated cleavage of the cytoskeleton liberates CD47 from these clusters, dispersing CD47 across the cell membrane. The dispersed CD47 molecules can no longer engage SIRPα, resulting in the loss of inhibitory signaling. Interestingly, dispersed CD47 may enhance efferocytosis through facilitating the binding of opsonins which allow for α_v_ integrins to bind to apoptotic cells [100,101]. The second signal is exposure of sufficient PtdSer on the apoptotic cell surface. A study involving the clearance of apoptotic Jurkat cells reveal that macrophages recognized these cells only when a critical threshold of surface PtdSer was reached, although this level was found to be much lower than the level of PtdSer typically exposed during apoptosis [102]. After these two conditions are met, efferocytic receptors can mediate engulfment of the apoptotic cell. 

The T cell immunoglobulin mucin domain (TIM) family of receptors is capable of directly binding PtdSer [97,103,104,105], with this binding occurring via a cation-dependent WFND motif in their extracellular domain [106]. Three TIM receptors, TIM-1, -3, and -4, are capable of efferocytosis, although TIM-4 has the highest affinity towards PtdSer and is expressed more broadly and at higher levels than TIM-1 and TIM-3 [98,107,108]. While these receptors directly bind to PtdSer, it is controversial whether they are capable of independently mediating efferocytosis. In some models, TIM-4 plays a supporting role by stabilizing the binding of the efferocyte to the apoptotic cell, thus enhancing efferocytosis via other receptors such as MERTK [93,109]. Further evidence of TIM-4 acting in concert with MERTK was found when comparing MERTK- and TIM-4-deficient resident peritoneal macrophages in mice. TIM-4^−/−^ macrophages had reduced apoptotic cell binding capability, whereas MERTK^−/−^ macrophages exhibited wild-type levels of binding, but both knockouts lacked the ability to internalize apoptotic cells [93,98,104,109]. This has led to the suggestion that TIMs may be tethering receptors which stabilize apoptotic cell–efferocyte interactions without being directly involved in the subsequent engulfment of the apoptotic cell. This view is controversial, with at least one study demonstrating TIM-4-dependent uptake of apoptotic cells [110]. Clearly, efferocytic receptors function in concert with each other, but the nature of these interactions and how they are regulated remain largely unexplored.

Another important receptor is the brain-specific angiogenesis inhibitor (BAI-1) receptor, a G-protein-coupled receptor [111]. BAI-1 binds to PtdSer through its extracellular thrombospondin Type I repeats. BAI-1 is a multifunctional receptor, in that it is also capable of binding bacterial lipopolysaccharides [112,113]. BAI-1 knockouts have an impaired ability to clear apoptotic cells, although this effect is limited to the thymus, testes, and colon [112]. Interestingly, BAI-1 also uses its PtdSer-binding ability to support muscle development, with BAI-1 deficiencies leading to reduced myoblast fusion [5].

In addition to PtdSer-recognizing receptors, scavenger receptors (i.e., receptors which bind to polyanionic ligands such as oxidized lipids and unusually glycosylated proteins) can also act as efferocytic receptors. Stabilin-1 and Stablin-2 are examples of scavenger receptors that act as efferocytic receptors in some tissues [83,114,115]. These proteins bind to a large range of ligands, including calreticulin, bacteria, and advanced glycosylation end products, via their FAS1 and EGF-like repeats [114,116,117]. Stabilin-2 binds directly to PtdSer, where, along with efferocytic receptors such as α_v_β_5_ integrins, it mediates efferocytosis [115]. SCARF-1, previously known as “scavenger receptor expressed by endothelial cell 1” (SREC-1, SR-F1), is an ortholog to the *C. elegans* receptor CED-1 [85]. Unlike the receptors discussed thus far, SCARF-1 indirectly recognizes phosphatidylserine through the opsonin C1q [118,119]. SCARF-1′s importance is highlighted by knockout studies revealing impaired apoptotic cell uptake and increased rates of autoimmunity [85]. SCARF-1^−/−^ mice were predisposed to systemic lupus erythematosus (SLE) and autoimmune nephritis due to an increase in circulating autoantibodies. In addition to scavenger receptors, other receptors are occasionally coopted as efferocytic receptors. For example, LDL-receptor-related protein (LRP) recognizes calreticulin, an ER-resident protein that is exported during apoptosis, with LRP promoting efferocytosis in some tissues [53,112].

Arguably, one of the most important families of efferocytic receptors is the integrin family, as many efferocytic receptors require integrins as co-receptors to mediate the internalization of the target apoptotic cell [92,112,120,121]. Multiple integrins can recognize apoptotic cells and participate in efferocytosis, including α_v_β_5_, α_v_β_3_, and α_x_β_2_. Integrins recognize apoptotic cells via the PtdSer opsonin milk fat globule-EGF-factor 8 (MFG-E8) [92,119,122]. MFG-E8 binds to PtdSer through its C2 domain [122] but also binds to other phospholipids exposed on the cell surface during apoptosis, including phosphatidylethanolamine. MFG-E8 contains a canonical integrin RGD binding motif, through which it is recognized by many integrins, including members of the α_v_, β_2_, and β_5_ families [120,123,124]. Recently, we identified another efferocytic integrin–opsonin pair (α_x_β_2_ and soluble CD93 (sCD93) [87]) but how this integrin is regulated during efferocytosis and the ligand for sCD93 remain to be characterized.

## 3. Fork and Knife: The TAM Family Takes Center Stage

**Figure 3:**
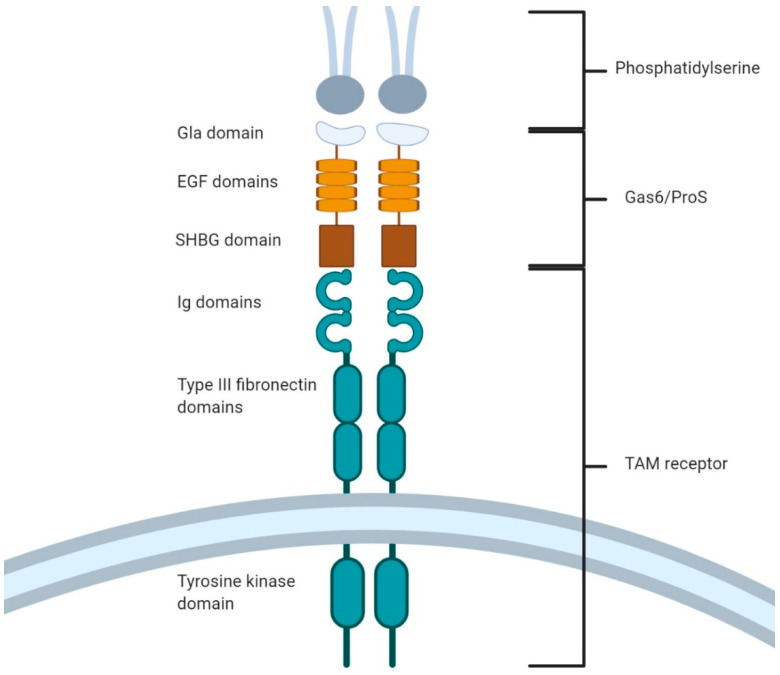
TAM (Tyro3, Axl, and MERTK) receptors are an important efferocytic receptor family. TAM receptors (blue) share a common structure of an extracellular domain comprising tandem Ig domains and Type 3 fibronectin domains, and an intracellular tyrosine kinase domain. Gas6 and ProS (brown) are opsonins that bind to PtdSer via their *N*-terminal Gla domain and to TAM receptors via their *C*-terminal SHBG domain. Opsonin binding dimerizes TAM receptors, resulting in the activation of the TAM kinase domains via cross-phosphorylation, thereby inducing downstream signaling.

While the above efferocytic receptors have known roles in the clearance of apoptotic cells, these receptors are often redundant to each other, with knockouts of individual receptors having a minimal effect on animal physiology, and with few mutations in these receptors having linkages to human disease [125]. The exception to this is the TAM (Tyro3, Axl, MERTK) family of efferocytic receptors [62,126]. TAM receptors are receptor tyrosine kinases characterized by an intracellular protein tyrosine kinase domain, a single transmembrane spanning domain, and an extracellular region comprising two extracellular Type 3 fibronectins and two Ig domains (Figure 3). Unlike the other known efferocytic receptors, mutations in TAM receptors—especially MERTK—have profound deleterious effects [62]. Inactivating mutations in MERTK causes retinitis pigmentosa, a form of progressive congenital blindness [127,128,129]. In these patients, uncleared apoptotic photoreceptor fragments accumulate within the eye and, over time, damage the retina [130]. This loss of MERTK function can also lead to progressive male infertility due to a loss of efferocytic activity in Sertoli cells in the testes [131]. Other mutations in MERTK and its opsonin, Gas6, are associated with increased susceptibility towards atherosclerosis and autoimmune disorders including multiple sclerosis and SLE [55,132].

All three TAM receptors recognize PtdSer indirectly through the opsonins Gas6 and Protein S (ProS), with other opsonins having been tentatively identified (Galectin-3, Tubby, and Tubby-like proteins) [133,134,135]. ProS is known for its role in coagulation in blood vessels and is found at much higher blood levels than Gas6 (25 µg/mL vs. 20–30 ng/mL) [136]. Both opsonins bind to TAM receptors in a calcium-dependent fashion and rely on their Gla domains for receptor activation [125,132,137,138]. Gla domains are rich in glutamate and receive post-translational modifications in a Vitamin K-dependent mechanism, resulting in a gamma carboxylation of Glu residues that is necessary for PtdSer binding and TAM receptor activation [62,139,140]. Phagocytic assays using Gla-deficient Gas6 and ProS led to a decreased ability to induce efferocytosis, with no change in the binding affinity of the opsonins for the TAM receptors [141]. Interestingly, Gas6 is capable of binding and activating TAM receptors in the absence of PtdSer, although it is unclear if this activation occurs at physiological levels of Gas6 [82]. Axl has the highest affinity for Gas6, leading to a constitutive association between the two proteins; indeed, circulating levels of free Gas6 are largely dependent on the degree of Gas6 sequestration by Axl [82,142]. In contrast, ProS can only be engaged by TAM receptors after binding and oligomerization on PtdSer-rich membranes. This oligomerization allows ProS monomers to auto-oxidize and form disulfide bridges with other ProS proteins, forming stable dimers that allow ProS to bind and activate TAM receptors [82,136,143]. This likely serves as a regulatory measure to prevent circulating monomeric ProS from activating TAM receptors, while also increasing the avidity of ProS interactions with TAMs. Gas6 and ProS also differ in the TAM receptors they ligate. MERTK is capable of binding to both opsonins, Axl only binds Gas6, and Tyro3 only binds ProS [82,125]. Other TAM ligands have been tentatively identified, thus explaining why knockouts of ProS and Gas6 do not result in complete loss of TAM function [132]. Tubby and Tulp-1 have been found to be MERTK ligands that interact with MERTK through their *N*- terminus [135]. Galectin-3 is another novel MERTK/Tyro3 ligand, but the domain used by Galectin-3 to interact with TAMs remains undefined [134,144]. All three of these opsonins are capable of promoting apoptotic cell clearance.

Although the TAM receptors belong to the same family, the roles they play in efferocytosis are not interchangeable. Indeed, the expression profiles of TAM receptors are highly tissue- and cell type-tropic. Dendritic cells primarily express Axl and Tyro3, whereas bone marrow-derived and tissue-resident-derived macrophages predominantly express MERTK [82,89]. Transcriptionally, MERTK and Axl/Tyro3 are inversely regulated, with stimuli that upregulate MERTK downregulating Axl and Tyro3, and vice versa [82,89,125,145]. Nevertheless, MERTK is still considered the primary TAM receptor for efferocytosis as Axl and Tyro3 knockouts have a minimal phenotype compared with MERTK knockouts [89,125]. On its own, Tyro3 is capable of apoptotic cell clearance but, as it is minimally expressed on efferocytic immune cell types such as macrophages, its physiological role appears to be minimal. On the other hand, Axl and MERTK play a more prominent role in apoptotic cell recognition, albeit in different situations. A study looking at the expression patterns between dendritic cells and macrophages revealed that dendritic cells express more Axl than MERTK, whereas macrophages primarily express MERTK [89]. In both cell types, PtdSer is recognized through Gas6, but only macrophages can recognize PtdSer via ProS due to their high expression of MERTK. Axl’s engagement in dendritic cells results in signaling that promotes an augmented inflammatory response and minimal efferocytosis [146]. For example, Zagórska *et al*. determined that the inflammatory response driven by LPS-mediated activation of Toll-like receptor 4 (TLR4) was magnified by Axl signaling, thereby bolstering the resulting inflammatory response and driving the polarization of resident tissue macrophages to an inflammatory (M1) phenotype [82]. This amplification of inflammation only occurred in the presence of low concentrations of PtdSer, suggesting that this pathway serves to limit the anti-inflammatory effects of efferocytosis in sites containing both apoptotic cells and pathogens. These sites contain apoptotic cells but would require ongoing inflammation to clear the pathogen. In the absence of LPS stimulation, Axl can serve as an efferocytic receptor and elicit anti-inflammatory responses [82]. In contrast, MERTK signaling is potently anti-inflammatory, largely through inducing the expression of Suppressor Of Cytokine Signaling 1 (SOCS1) and SOCS3. SOCS1 and -3 inhibit cytokine-induced JAK/STAT signaling, thereby reducing the expression of inflammatory cytokines and promoting the polarization of macrophages towards anti-inflammatory and highly efferocytic M2-like states [147].

TAM receptors rely on crosstalk with other efferocytic receptors for productive efferocytosis [121,146]. Integrins are particularly important for TAM function, notably α_v_β_3_ and α_v_β_5_ integrins, which are required for productive MERTK-driven efferocytosis in retinal pigment epithelial cells [121]. Indeed, activation of the MERTK kinase domain drives focal adhesion kinase (FAK) phosphorylation and activation, allowing FAK to be recruited to the cytoplasmic tail of α_v_β_5_ integrin. This enables the synergistic activation of Rac1, thus driving the cytoskeletal reorganization required to engulf an apoptotic cell [120,121,148]. The extent to which other integrins can be regulated by MERTK and the role of integrins in the function of the other TAM receptors remain largely unexplored.

## 4. Time to Dine: Engulfment of the Apoptotic Cell

Contact between an efferocyte and its target is initially limited to the small number of efferocytic receptors present where the efferocyte first contacts the apoptotic cell. The initial engagement of the apoptotic cell induces the recruitment of other efferocytic receptors into a larger synapse between the efferocyte and the apoptotic cell [61,124,149]. This mirrors pathogen phagocytosis, where a structured phagocytic synapse is formed, involving the organized distribution of receptors that cooperatively orchestrate phagocytosis [150,151]. The phagocytic synapse is comprised of a central region bearing phagocytic receptors surrounded by a ring of integrins, with the integrins forming a diffusion barrier that excludes inhibitory receptors such as the phosphatase CD45 from the interior of the synapse [152]. This enables productive phagocytic receptor signaling, driving the formation of a cup-like structure that eventually envelops the pathogen, resulting in its internalization into a plasma membrane-derived vacuole [153]. A similar process likely occurs during efferocytosis, with tethering receptors such as TIM-4, signaling receptors such as TAMs, and integrins coordinating to activate and organize the signaling pathways and cellular processes necessary for engulfment of the apoptotic cell. Whether this “efferocytic synapse” is similar in structure and function to the phagocytic synapse has not yet been explored but, given that both phagocytosis and efferocytosis rely on much of the same signaling and cellular processes to engulf their respective targets, it is likely that their synapses are also similar in structure and function.

**Figure 4:**
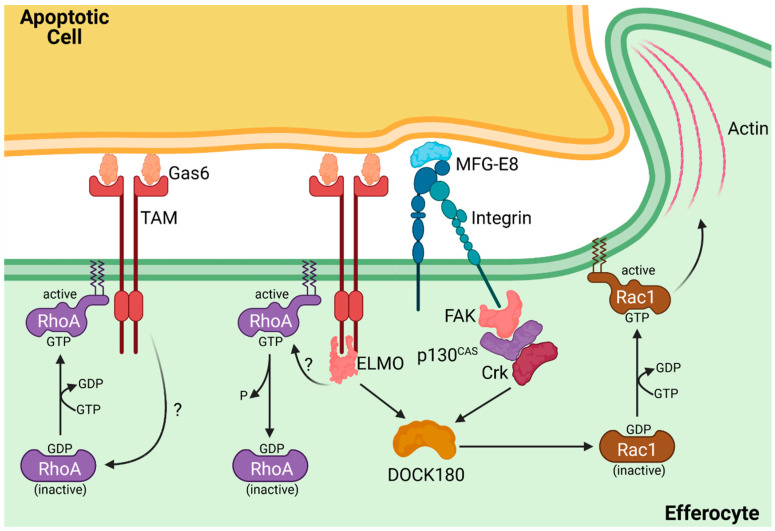
Rac1 and RhoA in efferocytic receptor signaling. Engagement of efferocytic receptors induces the initial recruitment of active (GTP-bound) RhoA to the membrane (left), which produces an initial inhibitory signal. Sufficient efferocytic receptor signaling induces the GTPase activity of RhoA, returning it to the inactive (GDP-bound) state, enabling efferocytic synapse formation (middle). The combined signaling of efferocytic receptors and integrins converges on DOCK180, which induces the activation of Rac1. Rac1 then mediates actin polymerization, which extends the efferocyte membrane around the apoptotic cell (right).

The dynamics behind apoptotic cell engulfment appear similar to conventional phagocytosis from a mechanical standpoint, starting with the formation of lamellipodia at the site of efferocyte–apoptotic cell contact [61,151,153]. These lamellipodia coalesce into an efferocytic cup, a ring-like structure partially enveloping the target cell. The leading edge of the cup extends around the apoptotic cell, leading to its internalization into a plasma membrane-derived organelle termed the efferosome. This membrane extension process is dependent on the manipulation of F-actin, which is regulated by Arp2/3 and Rho GTPases [154,155,156]. Actin polymerization pushes the edge of the efferocytic cup around the apoptotic cell, while actin depolymerization at the base of the cup allows for the nascent efferosome to enter the cytosol. In conventional phagocytosis, signaling through Fcγ receptors and other phagocytic receptors activates the canonical phagocytic signaling pathway, which converges on the activation of the Rho GTPases Rac1 and RhoA, which are GTPases also activated during efferocytosis (Figure 4) [154,156,157,158,159,160]. Both RhoA and Rac1 are GTPases: proteins which are activated by the exchange of GDP for GTP in their active sites [161]. In their active GTP-bound form, these proteins interact with effectors that regulate processes such as actin polymerization and the bundling of actin into stress fibers. These GTPases are inactivated by their intrinsic GTPase activity, hydrolysing the bound GTP into GDP. GDP–GTP exchange and induction of GTPase activity are, respectively, controlled by guanine exchange factors (GEFs) and GTPase-activating proteins (GAPs), with many GEFs and GAPs regulated by efferocytic and phagocytic receptor signaling. Genomic and structural analysis of Rac1 revealed the potential homology with CED-10, which, in both mammals and *C. elegans*, coordinates the actin cytoskeleton [162,163]. Analysis in *C. elegans* identified two evolutionarily conserved pathways that activate Rac1 during efferocytosis [157,162,163,164,165]. One of the pathways involves the CED-2/CED-5/CED-12 complex, for which the mammalian equivalent is the CrkII/ELMO/Dock180 complex [165]. Another pathway in Rac1 activation involves the CED-1/CED-6/CED-7 complex, which, in mammals, is equivalent to the paralogs LRP-1/GULP/ABCA1 [162,166]. The ELMO/DOCK180 complex and GULP protein are GEFs that activate Rac1 [157,164,167,168]. CrkII serves as an adaptor protein, binding to phosphorylated tyrosine residues in active receptors via its Src-homology 2 (SH2) domain, linking these receptors to the proline-rich region of the ELMO/DOCK180 GEF via its SH3 domains [165].

It has been proposed that tethering receptors such as TIM-4 cannot directly activate Rac1 but instead recruit integrins that bind to the apoptotic cell and subsequently induce Rac1 activation [110,121]. This model was proposed by Park *et al*., where, in epithelial cells, deletion of the TIM-4 intracellular domains had no impact on its ability to induce efferocytosis, suggesting that TIM-4 did not participate in efferocytic signaling [105]. However, later work by Flannagan *et al*. in macrophages demonstrated that TIM-4 could directly activate β_1_ integrins via signaling through Src family kinases (SFKs) and FAK [110]. This controversy remains unresolved, and it is unclear whether these differences are due to cell-type differences, the availability of different integrins, or other factors. Analysis of MERTK determined that SFKs and FAKs are required for efferocytosis via recruitment of α_v_β_5_, which, via the p130^CAS^/Dock180/Elmo complex, activates Rac1 (Figure 4) [121].

While TIM-4 and TAM receptors require integrins as co-receptors, other efferocytic receptors appear to be able to mediate engulfment independently. Upon binding to PtdSer, BAI-1 forms a trimer with ELMO and Dock180 to activate Rac1 [111]. BAI-1 lacking the extracellular (PtdSer-binding) domain is incapable of activating Rac1, as is BAI-1 lacking the intercellular ELMO/Dock180-binding domain. This shows that in BAI-1, PtdSer engagement is sufficient to activate Rac1 and mediate the resulting actin-driven engulfment of the apoptotic cell. Stabilin-2 is also capable of direct Rac1 activation via GULP adaptor protein-binding, which, via its PTB domain, can bind to the NPXY motif in the cytoplasmic tail of Stabilin-2, with GULP then activating Rac1 [168]. However, even with this capability to directly activate Rac1, Stabilin-2 has also been shown to recruit α_v_β_5_ via an extracellular bridge formed between the Stablin-2 fasciclin 1 domains and the integrin, suggesting that Stabilin-2 may require assistance from integrins for efficient efferocytosis [168].

The activation and role of RhoA in efferocytosis is not as well understood (Figure 4). FRET analysis of RhoA and Rac1 activation during efferocytosis determined that RhoA was present and active during the initial contact between the efferocyte and the apoptotic cell but quickly diminished following Rac1 activation and the onset of the formation of an efferocytic cup [159]. If inhibited, this RhoA activity led to uncontrolled efferocytosis. This accelerated efferocytosis was not dependent on CD47, suggesting that RhoA is involved in setting the threshold of PtdSer, which must be detected on an apoptotic cell before efferocytosis is induced [70]. Consistent with RhoA acting as a negative regulator of efferocytosis, statin treatment—which, in addition to lowering cholesterol levels, also reduces prenylation of RhoA—improves efferocytosis and patient outcomes in obstructive pulmonary disease [169].

## 5. Digesting the Apoptotic Meal: Vesicular Trafficking of Apoptotic Cells

The degradation of apoptotic cells following their engulfment occurs via two novel pathways. The first is LC3-associated phagocytosis (LAP) [47,170,171]. LAP involves the recruitment of LC3 onto the efferosomal membrane, which, in turn, recruits the autophagic machinery [170]. This allows the autophagy pathway, which is normally used to recycle damaged organelles, to mediate the degradation of the apoptotic cell. As this pathway is primarily homeostatic in nature, it does not engage the same antigen presentation and inflammatory pathways engaged following pathogen phagocytosis [172]. Defects in LAP impair the degradation of apoptotic cells by macrophages and increase the expression of inflammatory cytokines [171]. Not all apoptotic cells are degraded by LAP, with several studies identifying the canonical regulators of pathogen phagocytosis—Rab5 and Rab7—recruited to efferosomes independently of the markers of LAP such as LC3 [173,174]. Rab5 and Rab7 mediate the fusion of endosomes and lysosomes with the efferosome, thereby delivering the hydrolytic enzymes that degrade the apoptotic cell [175,176]. This same pathway is used for the degradation of pathogens; in this context, this pathway terminates in the formation of an MHC II loading compartment in which pathogen-derived antigens are loaded onto MHC II for presentation to the adaptive immune system [177,178]. This terminal step does not occur during efferocytosis; instead, the GTPase Rab17 is recruited to the efferosome, where it directs the degraded apoptotic cell materials out of the efferosome and into the recycling endosome, thereby limiting the presentation of apoptotic cell-derived antigens [179,180].

The efferocyte faces a significant metabolic burden following degradation of an apoptotic cell, as the engulfment of a single apoptotic cell represents a doubling—or more—of the macromolecular content of the efferocyte. Sterols represent the largest metabolic burden faced by efferocytes, with efferocytosis often followed by a reprogramming of the efferocyte metabolism to better process these materials [166,181,182]. Key to sterol processing is the upregulation of the ABCA1 transporter [166,182,183]. Sterols from the apoptotic cell are recovered within the efferosome by the sterol carriers NPC1 and 2, which transport the sterols to cytosolic carriers [184,185]. These carriers then deliver the sterols to other cellular membranes, with ABCA1 transferring the sterols from the inner leaflet of the plasma membrane to extracellular high-density lipoprotein (HDL) complexes [166,181]. HDL, via the circulation, then delivers this cholesterol to the liver, where it can be exported as bile salts [186]. The loss of ABCA1 function causes Tangier disease, which is characterized by low HDL plasma levels. Patients with Tangier disease are also at higher risk of atherosclerosis, likely due to a lack of efferocytic function in arterial tissue [181,187]. The upregulation of ABCA1, as well as other pro-efferocytic changes in efferocyte metabolism, is regulated by the nuclear receptors PPAR-γ and liver X receptor [63,166,188].

In addition to processing the engulfed apoptotic cell, efferocytes also manage tissue homeostasis at the sites of efferocytosis. By removing apoptotic cells prior to secondary necrosis, efferocytosis prevents the induction of inflammation. However, efferocytes not only prevent this induction of inflammation but also actively promote a pro-resolving and anti-inflammatory response. MERTK signaling, in addition to driving engulfment of the apoptotic cell, also suppresses inflammatory signaling and promotes the expression of anti-inflammatory cytokines [63,147,189,190]. The former occurs via MERTK-mediated Akt phosphorylation, which inhibits GSK3β, thereby limiting inflammatory signaling via pathogen-recognizing TLRs [189,190,191,192]. This same pathway inhibits NF-κB nuclear translocation, further preventing the expression of inflammatory cytokines following TLR signaling. MERTK, in cooperation with IFNα receptor (IFNAR), activates STAT1, which, in-turn, promotes the expression of additional MERTK [147,189,192]. The IFNAR/STAT1 signaling pathway also induces the expression of SOCS1 and 3. SOCS1 and SOCS3 inhibit inflammatory cytokine signaling by competing with JAKs for receptor binding, and by recruiting ubiquitin ligases to active cytokine receptors in order to ubiquitinate and degrade signaling molecules recruited to the receptor [192,193,194]. SOCS function is a fundamental component of MERTK-mediated suppression of inflammation, as mice lacking a functional SOCS1 gene possess a similar autoimmune phenotype to MERTK knockout mice and cannot inhibit TLR signaling or NF-κB activation in a MERTK-dependent manner [55,192,195,196]. Combined, the degradation pathways, metabolic reprogramming, and anti-inflammatory signaling used during efferocytosis ensure that apoptotic cells are removed in a fashion which preserves tissue homeostasis and limits immunogenicity. For a more detailed review of these anti-inflammatory and pro-healing mechanisms, see the recent review by Doran *et al*. [197].

## 6. The Other Menu: Necroptosis, Pyroptosis, and Ferroptosis

Apoptosis is not the only form of programmed cell death, with necroptosis, pyroptosis, and ferroptosis representing three other known programmed cell death pathways in metazoans. Necroptosis is an inflammatory form of cell death which was originally described as a “backup” to apoptosis that occurred when cell death stimuli were received in the absence of caspase signaling (e.g., in response to pathogens encoding caspase-inhibiting toxins) [198]. More recent evidence indicates that necroptosis may serve a broader role in controlling viral infection by inducing cell death in response to the activation of virus-recognizing TLRs in non-immunological cell types [199]. During necroptosis, activation of RIPK1 and RIPK3 by cell death receptors or TLRs induces the polymerization of MLKL in the mitochondria and plasma membrane. MLKL polymerization forms pores, which permeabilize these organelles, leading to cell rupture and the release of inflammatory cytosolic contents [200]. Pyroptosis is similar to necroptosis, in that it is a lytic form of inflammatory death induced by the presence of pathogens but, unlike necroptosis, pyroptosis is activated by intracellular pathogens and requires caspase activity. In pyroptosis, the activation of inflammasomes by cytosolic pathogen-derived molecules induces the activation of caspase-1, -4, and -5. These cleave and activate the inflammatory cytokines IL-1β and IL-18, as well as the pore-forming protein GSDMN. GSDMN pores in the plasma membrane allow for secretion of the active cytokines but can also lead to the swelling and lytic death of the cell [201,202,203]. Ferroptosis occurs when the cellular antioxidant machinery fails, leading to the accumulation of lipid peroxides. This form of cell death requires the accumulation of iron, which catalyzes lipid oxidation [204]. Ferroptosis most often occurs following loss of GPX4 activity, a peroxidase which normally reverses oxidative damage to lipids, often in response to chemotherapeutic and anti-rheumatic drugs [205]. Ferroptosis can also be driven by the disruption of iron homeostasis, such as that which can follow kidney injury [206]. The cell death mechanism of ferroptosis is not well understood but may involve permeabilization of the plasma membrane by the accumulation of oxidized lipids.

How cells that die through these pathways are cleared is not well elucidated, but it appears that many of the same mechanisms used for the clearance of apoptotic cells are used for the clearance of these cells. The cell lysis which occurs during necroptosis, pyroptosis, and ferroptosis releases the same nucleotide “find-me” signals as are released by pannexin channel cleavage during apoptosis [39,207]. Similarly, the ATP-dependent flippases and floppases that maintain the polarized distribution of PtdSer on the plasma membrane become inactive following the loss of cellular energetics, leading to the exposure of the “eat-me” signal PtdSer on the cell surface [208,209]. However, these non-apoptotic cell death pathways do not efficiently reduce “don’t-eat-me” signaling through CD47 signaling, leading to inefficient engulfment that can be ameliorated by resolvin-mediated downregulation of RhoA in the efferocyte [210]. Consistent with the efferocytic pathway being used to clear cells which die via non-apoptotic pathways, the engulfment of cells which die through these non-apoptotic pathways depends on the same opsonins (MFG-E8) and receptors (TIM-4) as efferocytosis, with TAM-derived anti-inflammatory signaling also limiting inflammation following engulfment of these cells [207,211]. To our knowledge, the maturation pathway used to degrade pyroptotic, necroptotic, and ferroptotic cells has not been explicitly investigated. This is an area of some interest, as it is currently unclear if these cells are degraded through the non-immunogenic pathway used to process apoptotic cells versus the immunogenic pathway used to clear pathogens, with the microbicidal nature of these “alternative” cell death pathways suggesting that the immunogenic maturation pathway may be used.

While the removal mechanisms of these non-apoptotic cell death pathways appear to be the same as those used to clear apoptotic cells, the immunological outcome of these cell death processes is vastly different. As discussed above, apoptosis utilizes multiple pathways to produce anti-inflammatory and non-immunogenic outcomes. Pyroptosis and necroptosis are cell death pathways which have explicitly evolved to induce inflammation, either through the deliberate release of inflammatory cytosolic contents (necroptosis) or through the co-production of inflammatory cytokines during cell death (pyroptosis) [212,213,214]. Indeed, these pathways are important for the clearance of some pathogens, including *K. pneumoniae*, which actively suppresses inflammatory cell death in order to limit inflammation and its clearance from infected neutrophils [209,215,216,217]. The use of these “alternative” cell death pathways is important for the elimination of many pathogens and has recently been reviewed by Zheng *et al*. [217]. Ferroptosis is not as well understood but appears to also be highly inflammatory [204]. Thus, while the clearance of apoptotic, necroptotic, pyroptotic, and ferroptotic cells appears to occur via a conserved cell-clearance mechanism, the specific cell death pathway used by a cell is what dictates the resulting immunological outcome.

## 7. Spoiling the Meal: Efferocytosis in Disease

As alluded to throughout this review, defects in efferocytosis have been associated with autoimmunity and chronic inflammation, largely through the release of inflammatory cytosolic contents and autoantigens during secondary necrosis [1,57,64,218]. While many inflammatory and autoimmune diseases have been associated with failed efferocytosis, these diseases share many mechanistic similarities that link failed efferocytosis to disease onset and progression. As such, for the sake of brevity, we will focus on the role of efferocytosis in multiple sclerosis, atherosclerosis, and cancer, through the lens of TAM receptors.

Multiple sclerosis is an autoimmune disease in which macrophages, microglia, B lymphocytes, and self-reactive T lymphocytes target the myelin sheath that insulates neurons [219,220]. The resulting demyelination abrogates neuronal transmission, thereby impairing CNS and motor function [221]. MERTK has been implicated in the pathogenesis of multiple sclerosis: patients with multiple sclerosis tend to have lower MERTK expression in the brain than healthy controls, and a number of single nucleotide polymorphisms in MERTK have been associated with multiple sclerosis [220,222,223]. There is also a decrease in Gas6 expression in the brains of patients with multiple sclerosis [224,225]. This loss of MERTK and its opsonin impairs the efferocytosis of myelin in these patients—a normally homeostatic process needed to maintain a healthy myelin sheath [226]. While the role of MERTK in this process is not completely elucidated, it is likely that the inability to properly clear aging myelin results in local inflammation, inducing an immunological response in the resident microglia and macrophages, with the free myelin subsequently activating myelin-reactive T cells [225]. Indeed, in a study using MERTK-KO microglia in a cuprizone model, microglia exposed to myelin debris expressed more IFNγ, resulting in decreased microglial activation and phagocytosis [227]. This created a feedback loop that further exacerbated the accumulation of myelin debris and further prevented remyelination [227]. Fortunately, this deficiency in efferocytic clearance is treatable with recombinant TGF-β, which restored expression of MERTK and Gas6 to basal levels in multiple sclerosis patients [224,225,228,229]. The benefits of TGF-β therapy extend beyond upregulating MERTK, as TGF-β also suppresses autoreactive T lymphocytes and promotes remyelination of neurons. Similarly, the use of the PPAR-γ agonist, pioglitazone, improves monocyte efferocytic function in multiple sclerosis patients, likely through the upregulation of MERTK and other efferocytic mediators [230].

Atherosclerosis is a cardiovascular disease involving the accumulation of fatty plaques along the artery endothelium, restricting blood flow [57,64,231]. These plaques are prone to rupture, which can lead to a heart attack or stroke. Atherosclerosis is a product of failed efferocytosis, wherein cholesterol loading of cardiac macrophages suppresses their efferocytic capabilities while driving their differentiation into highly inflammatory foam cells [57,232,233]. The stress of cholesterol loading eventually leads these foam cells to apoptose, but because efferocytosis is defective within the plaque, these apoptotic cells are left uncleared and eventually undergo secondary necrosis. This necrosis is highly inflammatory, driving the recruitment of additional macrophages, which then undergo the same cholesterol loading and apoptosis [187,218]. Ultimately, this results in a highly inflamed plaque with a core of necrotic foam cells and cell-free lipids, surrounded by a fibrotic capsule and infiltrated by inflammatory (M1-polarized) macrophages. Similar to multiple sclerosis, polymorphisms in MERTK and Gas6 are also associated with atherosclerosis [234,235]. Interestingly, wild-type MERTK is susceptible to cleavage by the metalloprotease ADAM17, with the resulting MERTK fragments being unable to elicit efferocytosis. Work from Cai *et al*. demonstrated that MERTK cleavage not only reduces efferocytosis but also promotes the formation of the necrotic core within the atherosclerotic plaque [218]. Expression of a cleavage-resistant MERTK mutant in this model prevented MERTK cleavage, reduced disease burden, and led to an increase in production of pro-resolving lipid mediators through the activation of 5-lipoxygenase. Strategies for treating atherosclerosis through restoring efferocytosis in the plaque have been explored and show promise in the treatment of the cardiovascular disease. For example, therapies blocking the “don’t-eat-me” receptor CD47 enhanced efferocytosis in the plaque, leading to plaque regression [236]. Moreover, lovastatin—a commonly used cholesterol-lowering drug—also improves efferocytic clearance of apoptotic cells by reducing the activity of RhoA in cardiac macrophages [169,237].

In cancer, it is not defective efferocytosis that drives pathology; rather, the unwanted activation of the efferocytic system allows cancers to persist and overcome immunological clearance. Many cancers have been found to express MERTK or another TAM receptor, through which they clear dying tumor cells [238,239]. This enhances cancer growth through at least two mechanisms: the production of anti-inflammatory cytokines, many of which act as growth factors, and via sequestration of tumor antigens [238]. Combined, these limit T cell activation against tumor-derived antigens, thereby reducing anti-tumor immune responses. This is further facilitated by tumor-associated macrophages, which exhibit a highly efferocytic and immunoregulatory phenotype, and which express high levels of MERTK that further enhance the non-immunogenic clearance of apoptotic cancer cells [240,241]. Lastly, MERTK itself can act as an oncogene, with mutations in its kinase domain having a direct oncogenic effect [242,243]. Several inhibitors of the TAM receptor kinase domain have been developed for the treatment of cancer, with some showing good efficacy in pre-clinical models [244,245,246]. While the exact effect of these inhibitors on the tumor has not been explored in detail, it is thought that they work by increasing secondary necrosis within the tumor microenvironment by reducing efferocytosis. This allows for the induction of a pro-inflammatory response which is amplified by the blockage of TAM-mediated anti-inflammatory cytokine production, resulting in greater tumor killing and a decrease in tumor mass [245]. This concept was partially confirmed in a recent study by Zhou *et al*., wherein it was demonstrated that MERTK blockade led to danger-associated molecular pattern release from tumor cells undergoing secondary necrosis [247]. This induced a Type I IFN response in the tumor-associated macrophages, prompting them to initiate a pro-inflammatory response. Thus, targeting MERTK in cancer may not only increase tumor immunogenicity but may also augment immune checkpoint inhibitor therapies through increasing tumor inflammation.

The beneficial effect of MERTK inhibition appears to conflict with the need to engulf apoptotic cells for the purpose of presenting tumor-derived antigens to T cells, which is a critical step in generating a cytotoxic immune response against the tumor. The success of these early trials suggests that this is a not a limitation of this therapeutic approach, although the mechanisms allowing for efficient T cell activation in the presence of MERTK inhibition is unexplored. However, it was established that necrotic cells—presumably including uncleared apoptotic cells which subsequently die through secondary necrosis—are cleared by non-TAM receptors such as CLEC9A [248]. CLEC9A (also called DNGR-1) allows dendritic cells to engulf necrotic cells and then cross-present tumor-derived antigens on MHC I through a mechanism which ruptures the efferosome, thus allowing necrotic-cell antigens to be loaded onto MHC I in the endoplasmic reticulum [249,250]. Moreover, dendritic cells are known to preferentially utilize Axl for efferocytosis, with current MERTK inhibitors showing good selectivity for MERTK over the other TAM receptors [89,251], indicating that TAM-dependent uptake of tumor antigens by dendritic cells may be minimally affected by MERTK inhibition. Clearly, much remains to be discovered in the interplay of efferocytosis, tumor immune evasion, and tumor immunogenicity, but these early results indicate that targeting efferocytosis is a promising avenue of research for future cancer immunotherapies.

## 8. Conclusions

Efferocytosis is an immunoregulatory response in which apoptotic cells are phagocytosed by local phagocytes. This elicits a pro-resolving and anti-inflammatory response, promoting tissue homeostasis and averting the inappropriate inflammatory and autoimmune impacts of secondary necrosis. Apoptotic cells are usually recognized and cleared via recognition of PtdSer on their surface, with this PtdSer recognized by multiple efferocytic receptors with non-overlapping functions that cooperate in order to internalize the apoptotic cell. TAM receptors play a central role in this process and appear to coordinate much of the engulfment process, as well as eliciting a potent anti-inflammatory response. Although the signaling pathways associated with the actin cytoskeletal rearrangements and anti-inflammatory cytokine response have been identified, how these signals arise from the interplay between different efferocytic receptors has yet to be elucidated. Defects in efferocytosis are implicated in the pathobiology of autoimmune disorders and chronic inflammation, with defects in efferocytosis driving the release of inflammatory and antigenic cytosolic contents via secondary necrosis. On the other side of the coin, efferocytosis contributes to the immunosuppressive nature of the tumor microenvironment, primarily through promoting the formation of anti-inflammatory tumor-associated macrophages and through antigen sequestration. These observations indicate that targeting efferocytosis is likely to be a productive approach in developing new therapies for inflammatory disease, autoimmunity, and cancer.

## Figures and Tables

**Table 1:** Cell-Type Specific Expression of Major Efferocytic Receptors.

Cell Type	Receptors	Citations
Tissue-Resident Macrophages ^1^	MERTK ^2^, Axl ^2^, TIM-4, Stabilin 1, Stabilin 2 ^2^, α_v_β_5_, α_x_β_2_, CD36, SCARF-1, LRP-1	[66,67,68,69,70,71,72,73,74,75,76]
Bone Marrow Derived Macrophages	MERTK ^2^, Axl ^2^, TIM-4, Stabilin 1, Stabilin 2, α_v_β_5_, α_v_β_3_, α_x_β_2_, CD36, SCARF-1, LRP-1	[66,67,77,78,79,80,81,82,83]
Dendritic cells	Tyro3, Axl, MERTK ^2^, TIM-4 ^2^, Stabilin 1, α_v_β_5_, SCARF-1	[80,84,85,86,87]
Microglia	Axl, MERTK, TIM-4, Stabilin 1, α_v_β_5_, α_v_β_3_, BAI-1	[66,79,88,89,90,91,92]
Kidney Tubule Epithelial Cells	TIM-1	[93]
Retinal Pigment Epithelium Cells	MERTK, α_v_β_5_	[94,95]
Myoblasts ^3^	BAI-1	[96]
Osteoclasts	BAI-1, TIM-4, Stabilin 1	[97]

^1^ Tissue-resident macrophages are the predominant efferocyte in most tissues. ^2^ Relative expression of these receptors can change based on extracellular stimuli. ^3^ BAI-1 expression in myoblasts mediates cell fusion; efferocytic myoblasts have not been reported.

## Data Availability

Not applicable.
